# The GNAO1-B Splice Variant Is the Predominant Isoform in Human Astrocytes and Localizes to Retraction Fibers and Migrasomes

**DOI:** 10.3390/cells14221755

**Published:** 2025-11-10

**Authors:** Egor A. Volovikov, Alina V. Davidenko, Elizaveta V. Emets, Anastasia S. Smirnova, Alexandra N. Bogomazova, Maria A. Lagarkova

**Affiliations:** 1 Lopukhin Federal Research and Clinical Center of Physical-Chemical Medicine of Federal Medical Biological Agency, Moscow 119435, Russiaabogomazova@rcpcm.org (A.N.B.); lagar@rcpcm.org (M.A.L.); 2 Federal State Budgetary Institution of Science “M.M. Krasnov Research Institute of Eye Diseases” (Krasnov Research Institute of Eye Diseases), Moscow 119021, Russia; 3 Centre for Regenerative Medicine, Medical Research and Education Institute, Lomonosov Moscow State University, Moscow 119234, Russia; 4 Faculty of Medicine, Medical Research and Education Institute, Lomonosov Moscow State University, Moscow 119234, Russia

**Keywords:** GNAO1, IPSC, astrocytes, retraction fibers, migrasomes, G-protein

## Abstract

GNAO1 is an alpha subunit of the G-protein complex involved in signal transduction in neurons. The G203R mutation in the GNAO1 gene arises recurrently de novo and causes epileptic encephalopathy and movement disorder. GNAO1 has two main isoforms, GNAO1-A and GNAO1-B, but their functional or expression differences are poorly understood. Molecular functions of GNAO1 are mainly studied in neurons, yet glial cells also express GNAO1 and participate in the pathogenesis of epilepsy. Here, we used human-induced pluripotent stem cell-based models to investigate the localization and expression of GNAO1 isoforms in astrocytes. We showed that in astrocytes, almost 100% of GNAO1 transcripts encoded GNAO1-B with very low GNAO1-A expression. We showed that there were no differences in localization between GNAO1-A and GNAO1-B, both in WT and G203R states. We also showed that GNAO1 localized in astrocytic retraction fibers and migrasomes, structures not previously described in this cell type. We showed that GNAO1-positive retraction fibers of neighboring cells provided cell-to-cell contacts and also provided calcium waves during astrocytic excitation. Overexpression of both GNAO1-A and GNAO1-B tends to lower calcium activity in astrocytes, with GNAO1-A providing the most severe impairment of activity. Our results demonstrate that astrocytes, in addition to neurons, should be used as a model for studying GNAO1-related disorders and that GNAO1 mutations should be evaluated in the context of both the GNAO1-A and GNAO1-B isoforms.

## 1. Introduction

The GNAO1 gene encodes the α-subunit of heterotrimeric G-protein complexes, which plays a crucial role in transducing signals from G-protein-coupled receptors (GPCRs). GNAO1 is one of the most abundantly expressed membrane proteins in the human brain [1,2]. Mutations in this gene have been associated with the onset of developmental and epileptic encephalopathies and movement disorders. Symptoms include epileptic seizures, hypotonia, dystonia, developmental delay, intellectual disability, ataxia, and other movement disorders [3,4,5]. More than 80 pathogenic variants of GNAO1 have been described to date [6]. The heterogeneity in clinical symptoms arising from mutations at various functional sites of the GNAO1 protein reflects its diverse molecular functions [6].

GNAO1 is mainly expressed in the cortex, striatum, and hippocampus but is present in almost every region of the brain [7]. One of GNAO1’s molecular functions is to regulate the synthesis of cAMP. After receiving a signal from a GPCR, GNAO1 changes its conformation, binds GTP, and dissociates from the heterotrimeric G-protein complex. In free form, it regulates the activity of transmembrane adenylyl cyclases, which generate cAMP [8]. GNAO1 is known to mediate inhibitory signaling of GABA, dopamine, and adenosine neurotransmitters [9,10]. Recently, it was shown that GNAO1 plays a role in early neurogenesis, where the G203R mutation leads to impairment in the formation of neural rosettes in human neuronal progenitors. Furthermore, GNAO1 is involved in organizing the cytoskeletal remodeling and functional polarity of neurons in the developing brain [11,12].

GNAO1 has two major splice forms: GNAO1-A (NM_020988.3) and GNAO1-B (NM_138736.3). They encode proteins of a similar length (354 amino acids (AAs)) and share highly similar AA sequences. GNAO1-A and GNAO1-B differ by only 20 AA, which are mostly located in a cluster of α-helices near the C-terminus of the protein and in their 3′UTR regions. Little is known about the functional differences between these isoforms. Although most pathogenic mutations are located in sequences shared by both isoforms or in regions specific to GNAO1-A, several pathogenic mutations have also been found in GNAO1-B-specific sequences [13].

Many mouse, drosophila, worm, and human cell models have been focused on the effects of GNAO1 mutations on neuron functions [11,12,14,15,16]. While glial cells also express GNAO1 at a significant level, only a few studies have investigated GNAO1 function in these cells. To our knowledge, only one study has focused on GNAO1 functions in Schwann cells, showing that GNAO1 overexpression in mice leads to reduced myelinating potential [17]. Astrocytes are another glial subtype that plays a crucial role in the metabolism of neurotransmitters, absorbing them from the synaptic cleft, maintaining the blood–brain barrier, and preventing neural inflammation processes [18,19,20]. Astrocytes also exhibit intracellular calcium waves that allow them to integrate multiple neurotransmitter signals from different synapses and to modulate synaptic activity and architecture [21]. These calcium waves result from GPCR activation and subsequent G-protein signalling. Astrocytes also participate in neuronal synapse functioning by releasing gliotransmitters that can modulate post- or presynaptic activity in neurons [22]. Impairment of astrocyte function is linked to epileptic seizure onset and development [23,24,25]. Here, we used an induced pluripotent stem cell (IPSC)-based model to identify the expression and localization pattern of GNAO1 isoforms in human astrocytes, both in wild-type and G203R states.

## 2. Materials and Methods

IPSC lines

In this study, we used three IPSC lines obtained from healthy donors and one IPSC line obtained from a patient with the G203R mutation in the GNAO1 gene. All lines were published previously (Table 1).

IPSC cultivation

IPSCs were cultured on hESC-grade Matrigel (Corning, Corning, NY, USA) matrix in mTesR1 medium (StemCell Technologies, Vancouver, BC, Canada), which was changed daily. Passaging was performed using 0.05% trypsin-EDTA (Gibco, Waltham, MA, USA). After IPSC passaging, the medium was supplemented with 5 μM of the selective inhibitor of rho-associated protein kinase (ROCK) Y-27632 (DC Chemicals, Shanghai, China) for 24 h.

Neuronal differentiation

Neurons were obtained as previously described [28]. Briefly, IPSCs were grown to full confluence and cultivated for 14 days in medium consisting of DMEM/F12 (Paneco, Moscow, Russia), 1x penicillin–streptomycin (P/S) (Paneco, Moscow, Russia), 1xGlutamax (Gibco, Waltham, MA, USA), 1xN2 supplement (Paneco, Moscow, Russia), 2% knock-out serum replacement (SR) (Gibco, Waltham, MA, USA), and SMAD-inhibitors 2 μM dorsomorphin (DC Chemicals, Shanghai, China), 10 μM SB431542 (DC Chemicals, Shanghai, China), and 1 μM LDN-193189 (DC Chemicals, Shanghai, China). After that, cells were passaged by Versene (Paneco, Moscow, Russia) solution to a Matrigel-coated surface in a 1:2 ratio with supplementation of 5 μM Y-27632 for 24 h. Then, cells were cultured for 14 days in medium consisting of a 1:1 mixture of DMEM/F12 and Neurobasal (Paneco, Moscow, Russia), 1xP/S, 1xGlutamax, 1xN2, 1xB27 (Paneco, Moscow, Russia), 2% SR, 20 ng/mL FGF2 (Miltenyi Biotec, Bergisch Gladbach, Germany), and 2 μM hedgehog pathway activator purmorphamine (DC Chemicals, Shanghai, China). Media were changed daily, and passaging was performed every 7 days with Versene solution in a 1:2 ratio on Matrigel-coated surface with supplementation of 5 μM Y-27632 for 24 h. Next, cells were seeded at a high density of 400.000/ cm^2^ on a Matrigel-coated surface in medium consisting of Neurobasal, P/S, 1xGlutamax, 1xB27, 20 ng/mL BDNF (Miltenyi Biotec, Bergisch Gladbach, Germany), 20 ng/mL GDNF (Miltenyi Biotec, Bergisch Gladbach, Germany), and 5 μM forskolin (DC Chemicals, Shanghai, China) and cultivated for 30 days. Medium was changed every 3 days. In RNA expression analysis, three separate differentiation replicates were performed for each donor.

Astrocyte differentiation

Astrocytes were obtained as previously described [29]. Briefly, IPSCs were grown to full confluence and cultivated for 14 days in medium consisting of DMEM-F12, 1xP/S, 1xGlutamax, 1xN2, 2% SR, 2 μM dorsomorphin, 10 μM SB431542, and 1 μM LDN-193189. After that, cells were passaged by Versene solution at a high density of 200.000/cm^2^ with supplementation of 5 μM Y-27632 for 24 h. This and consecutive passages were performed with 0.05% trypsin, and cells were seeded on Matrigel-coated surfaces every 3 days with a negative progression of cell-seeding density (less by 20.000 cells/cm^2^ for every next passage). Cells were cultivated for 6 days in medium consisting of DMEM/F12, 1xN2, 1x non-essential amino acids (NEAAs) (Paneco, Moscow, Russia), 1xP/S, 1xGlutamax, 1% FBS (Gibco, Waltham, MA, USA), 20 ng/mL EGF (Miltenyi Biotec, Bergisch Gladbach, Germany), and 20 ng/mL FGF2. Then, cells were cultivated for 6 days in medium consisting of DMEM/F12, 1xN2, 1xNEAA, 1xP/S, 1xGlutamax, 1% FBS, 20 ng/mL EGF, 10 ng/mL FGF2, and 20 ng/mL CNTF (Miltenyi Biotec, Bergisch Gladbach, Germany). Next, cells were cultivated for 6 more days in medium consisting of DMEM/F12, 1xN2, 1xNEAA, 1xP/S, 1xGlutamax, 1% FBS, 20 ng/mL EGF, and 20 ng/mL CNTF. Thereafter, cells were cultivated in medium consisting of DMEM/F12, 1xN2, 1xNEAA, 1xP/S, 1xGlutamax, 1% FBS, and 20 ng/mL CNTF. Medium was changed every 3 days. For RNA expression analysis, three separate differentiation replicates were performed for each donor.

Mycoplasma testing

All cell cultures were routinely tested for mycoplasma contamination every 7 days. DNA was extracted from cell suspensions, and the mycoplasma-specific sequence was amplified via PCR as described previously [27]. All cell lines used in the present study were mycoplasma-free.

RNA isolation and RT-qPCR

RNA was isolated using TRIzol reagent (Thermo Fisher Scientific, Waltham, MA, USA) according to the manufacturer’s protocol. cDNA was synthesized using an MMLV-reverse transcriptase kit (Evrogen, Moscow, Russia) according to the manufacturer’s protocol. RT-qPCR with TaqMan-like probes specific for GNAO1 isoforms was performed in a single tube with qPCR-HS mix (Evrogen, Moscow, Russia) according to the manufacturer’s protocol. Plasmids containing the full coding sequences of wild-type GNAO1-A and GNAO1-B were used as standards for quantification of the isoform copy number. RT-qPCR with SYBR-green detection was performed with qPCR-HS-SYBR mix (Evrogen, Moscow, Russia) according to the manufacturer’s protocol. Relative GNAO1-A and GNAO1-B expression was calculated as the absolute amount of the tested isoform divided by the combined amount of both isoforms. Table 2 lists the primers and probes used in this study.

Molecular cloning

To amplify the coding sequences (CDS) of GNAO1-A and GNAO1-B, we extracted RNA from neurons derived from IPSCs of the GNAO1 patient with the G203R mutation, synthesized cDNA using high-fidelity Magnus reverse transcriptase (Evrogen, Moscow, Russia), and performed amplification with specific primers (Table 2) and high-fidelity Q5-HS polymerase (NEB, Ipswich, MA, USA) according to the manufacturer’s protocol. The amplified CDS were ligated into PJET1.2/blunt vector (Thermo Fisher Scientific, Waltham, MA, USA). Allele identification and verification of the absence of amplification errors were performed through Sanger sequencing of the inserted fragments. The identified and verified CDS for GNAO1-A and GNAO1-B, both in WT and G203R states, were then cloned into lentiviral vector plasmids LeGo-G2 or LeGo-C2 (Addgene #25917 and #27339). To generate a lentiviral vector plasmid expressing alpha-tubulin fused with RFP, we cloned an RFP-tubulin CDS from the pTagRFP-tubulin vector (Evrogen, Moscow, Russia) to LeGo-G2 (Addgene #25917) in place of GFP. All cloned plasmid sequences were verified by Sanger sequencing.

Immunocytochemistry

For immunocytochemical staining, cells were cultured on Matrigel-coated 8-well glass-bottom chamber slides. At the start of the staining procedure, cells were washed with warm PBS (Paneco, Moscow, Russia) and fixed in 4% PFA for 15 min. They were then washed with PBS three times for 5 min each, permeabilized with 0.1% Triton X-100 (Biorad, Hercules, CA, USA) solution for 15 min, washed again with PBS, and incubated for 1 h at RT in a blocking solution consisting of PBS, 5% FBS, 5% goat serum, and 0.1% Tween-20 (Applichem, Darmstadt, Germany). Primary antibodies were added to the blocking solution according to the dilution table (Table 3) and incubated overnight at 4 °C. Next, cells were washed three times with PBS with 0.1% Tween-20 (PBST), and secondary antibodies diluted in PBST were added for 1 h at RT. Next, cells were washed three times for 5 min each with PBST, stained with a 200 ng/mL DAPI solution, and washed again with PBST. Cells were visualized under a confocal microscope (Olympus FV3000, Tokyo, Japan) or a widefield fluorescence microscope (Nikon Eclipse with DS-Qi-2 camera, Tokyo, Japan).

Western blotting

For protein extraction, cells were detached from the matrix with Versen solution and centrifuged for 5 min at 200 g (RT). The cell pellet was resuspended in RIPA buffer (Thermo Fisher Scientific, Waltham, MA, USA) and incubated for 1 h at 4 °C. The protein concentration was measured using a Pierce BCA kit (Thermo Fisher Scientific, Waltham, MA, USA), and samples were normalized to equal concentrations by adding RIPA buffer. The protein lysates were then mixed with 2× Laemmli buffer (Biorad, Hercules, CA, USA), incubated at 92 °C for 20 min, and loaded onto a polyacrylamide gel. Denaturing electrophoresis was performed using a Mini-PROTEAN Tetra Cell system (Biorad, Hercules, CA, USA) according to the manufacturer’s protocol. Transfer was carried out using the Trans-Blot Turbo semi-dry transfer system (Biorad, Hercules, CA, USA). The membrane was blocked with a milk-based blocking solution (Biorad, Hercules, CA, USA). The membrane was incubated with primary antibodies (Table 3) for 24 h at 4 °C, washed three times with PBST at RT, incubated with secondary antibodies (Table 3) for 1 h at RT, and washed three times with PBST at RT. Signal detection was performed using SuperSignal West Femto Maximum Sensitivity Substrate (Thermo Fisher Scientific, Waltham, MA, USA) according to the manufacturer’s protocol.

Lentivirus generation and infection

Third-generation lentiviral vectors based on the LeGo system were produced in the Phoenix cell line. Plasmid transfection was performed using GenJect-39 lipofection reagent (Molecta, Moscow, Russia) according to the manufacturer’s protocol. Lentiviruses were collected every 24 h for 3 days, filtered through a 0.45 µm filter, aliquoted, and stored at −70 °C until use. Lentiviral titer was calculated based on the mean number of GFP-positive HEK293 cells infected with serial dilutions of the viral stock and quantified using an Acea Novocyte flow cytometer (ACEA Biosciences, San Diego, CA, USA). Astrocyte infection was performed at a multiplicity of infection (MOI) of 5. During infection, cells were cultured for 24 h in medium supplemented with heat-inactivated FBS and 10 μg/mL polybrene (SantaCruz Biotechnology, Dallas, TX, USA). Table 4 lists the lentiviral vectors used in this study.

Golgi staining

The Golgi apparatus in live cells was stained with BDP-TR ceramide (Lumiprobe RUS, Moscow, Russia) according to the manufacturer’s protocol.

Confocal imaging

Microscopy of live and fixed cells was performed using a confocal microscope (Olympus FV3000). For live-cell imaging, laser intensity was set to a low level to minimize phototoxic effects on cells during continuous imaging. For calcium imaging, cells were treated with FluoriCa-8 AM (Lumiprobe RUS, Moscow, Russia) according to the manufacturer’s protocol and visualized with a confocal microscope at a frequency of 1 Hz for 15–30 min.

Calcium peaks analysis

Calcium peaks were analyzed with the Fluorosnapp application [30].

Statistical analysis

All statistically analyzed data were tested for normality using the Shapiro–Wilk test. An unpaired *t*-test was used to analyze the difference in total GNAO1 expression levels (relative to GAPDH) between neurons and astrocytes in the RT-qPCR experiments. For isoform-specific analysis, absolute amounts of GNAO1-A and GNAO1-B in the samples were calculated from a standard dilution curve. These values were summed to determine the “total GNAO1 expression,” and the percentage of each isoform relative to this total (isoform proportion) was calculated. The differences in GNAO1-A and GNAO1-B percentage values were assessed using the Wilcoxon test. Statistical analysis of the calcium imaging data was performed using the Wilcoxon test.

## 3. Results

### 3.1. Cell Model Generation

**Figure 1:**
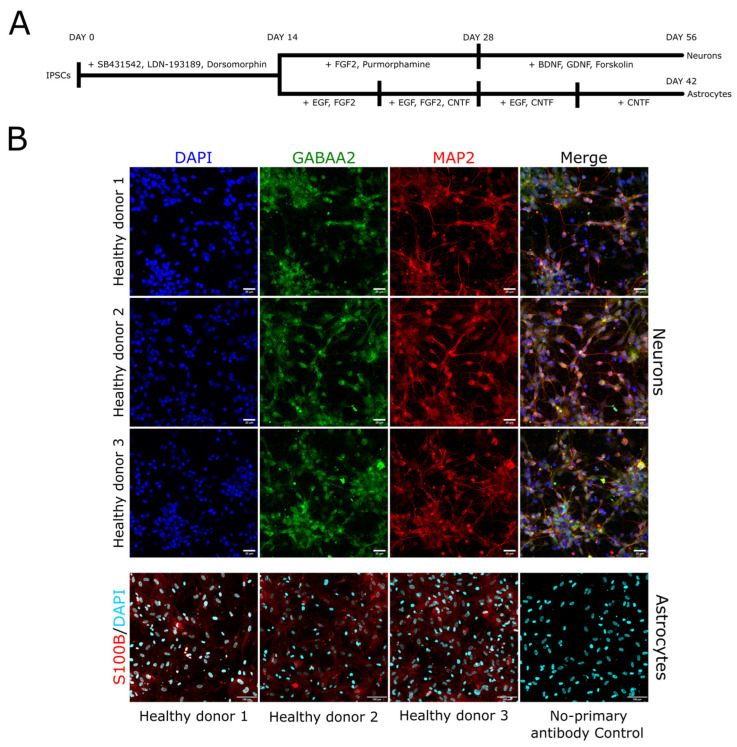
(**A**) Schematic representation of differentiation protocol for generating GABAergic neurons and astrocytes from IPSCs. Small molecules and proteins used are indicated above corresponding time period. (**B**) Immunocytochemical staining of the generated astrocytes and neurons. Neurons expressed the specific neuronal marker MAP2 (shown in red) and the GABAA receptor subunit alpha-2 (shown in green) (confocal imaging). Scale bar is 20 µm. Astrocytes expressed S100B, a specific astrocytic marker (confocal imaging, maximum intensity projection). Scale bar is 100 µm. DAPI nuclear staining is shown in blue or cyan.

From the IPSCs of three healthy donors using previously described protocols [28,29] based on dual-SMAD inhibition, we separately differentiated astrocytes and GABAergic neurons (Figure 1A). To prove the specificity and quality of differentiation, we performed immunocytochemical analysis. Astrocytes expressed the calcium-binding protein S100B (Figure 1B) and GFAP (supplementary Figure S1), which are widely used as specific astrocytic markers [31,32], and showed calcium wave oscillations and propagation (supplementary video S1). Neurons expressed the mature neuronal marker MAP2 [33], and most cells were positive for GABA-A receptor subunit alpha2 [34], allowing us to classify them as mature GABAergic neurons (Figure 1B). Some cells in the neuronal culture were positive for S100B or tyrosine hydroxylase (a marker of dopaminergic neurons [35]), yet their numbers were too low to significantly influence expression patterns (supplementary Figure S1). Therefore, the developed astrocytic and neuronal cultures represented appropriate models for the following studies on GNAO1 expression and localization.

### 3.2. Astrocytes Predominantly Expressed GNAO1-B Variant

**Figure 2:**
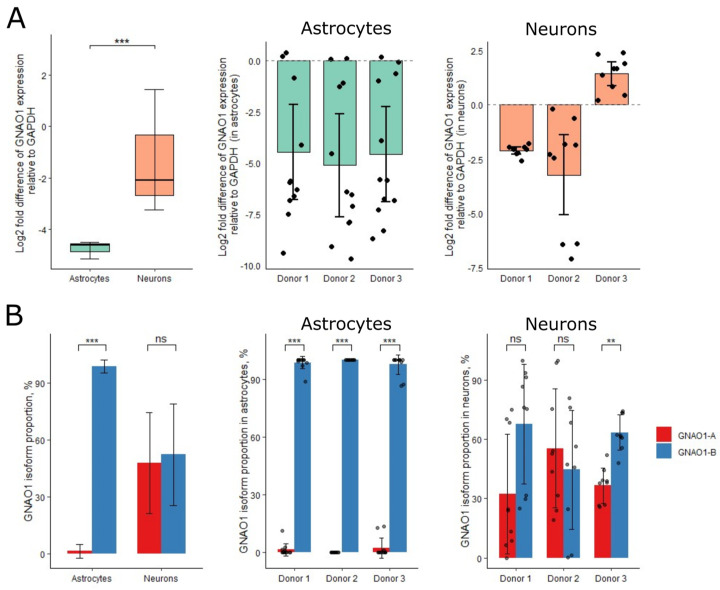
(**A**) Total GNAO1 expression fold difference compared to GAPDH in astrocytes and neurons (**left panel**), in astrocytes from individual donors (**middle panel**), and in neurons from individual donors (**right panel**). In left panel, whiskers represent 1.5 interquartile range of the data. In middle and right panels, whiskers represent standard deviation based on biological replicates (separate differentiation processes). Dots represent data distribution. (**B**) Isoform proportion of GNAO1-A and GNAO1-B in astrocytes and neurons (**left panel**), in astrocytes from individual donors (**middle panel**), and in neurons from individual donors (**right panel**). Whiskers represent standard deviation based on biological replicates (individual donors with three separate differentiation processes for the left panel and separate differentiation processes for middle and right panels). Dots represent data distribution. Significance levels: **—*p* < 0.01, ***—*p* < 0.001, ns— *p* > 0.05.

We analyzed total GNAO1 expression in astrocytes and neurons. The mean expression level of GNAO1 in astrocytes was lower than in neurons but remained significantly high (Figure 2A). We then used GNAO1-A- and GNAO1-B-specific TaqMan-like probes to investigate the expression of GNAO1 isoforms in astrocytes and neurons. Neurons expressed both GNAO1-A and GNAO1-B at nearly equal levels (47.8% and 52.3%, respectively; SD = 26.7%). In contrast, astrocytes exhibited strongly predominant GNAO1-B expression (98.8%, SD = 3.5%), with minimal GNAO1-A expression (1.2%; SD = 3.5%) (Figure 2B). The high SD in neurons raised a question about the stability of GNAO1 isoform expression and its dependence on the genetic background of each donor. Donors 1 and 2 showed no statistically significant difference in isoform proportion, though their mean pattern differed: GNAO1-B expression was higher in donor 1, while GNAO1-A expression was higher in donor 2. Only donor 3 exhibited a statistically significant difference (*p* < 0.01), with GNAO1-B being the most expressed isoform (63.5%; SD = 8.9%). In astrocytes, all three donors showed significant differences in GNAO1 isoform proportion, with GNAO1-B being the predominantly expressed isoform (minimum 97.7% expression) (Figure 2A).

### 3.3. GNAO1-A and GNAO1-B Showed No Difference in Intracellular Localization in Astrocytes

**Figure 3:**
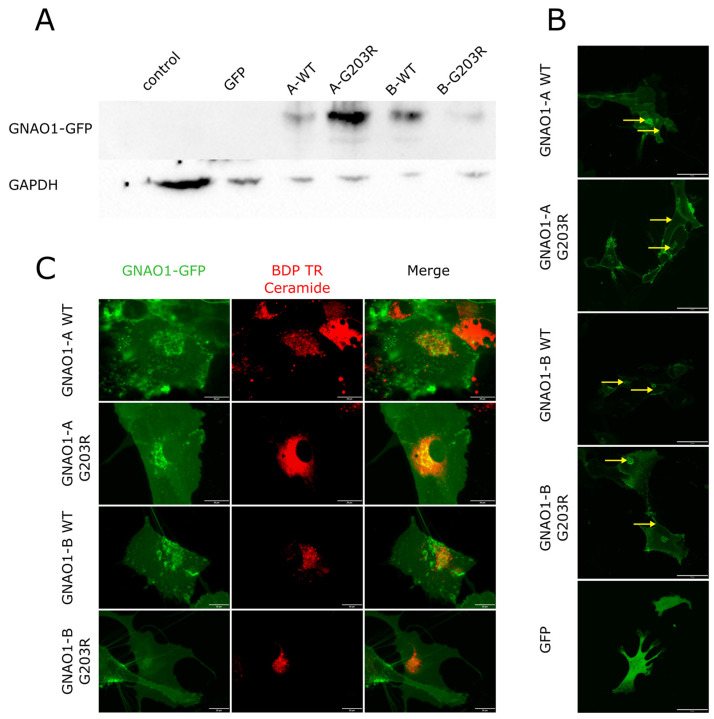
(**A**) Western blot analysis of uninfected or lentivirus-infected HEK293 cells. The upper panel shows GNAO1 expression; the lower panel shows GAPDH expression. (**B**) Live confocal imaging of astrocytes expressing GNAO1-GFP variants or GFP alone. Yellow arrows indicate GNAO1 localization on the cell membrane and in the Golgi apparatus. Scale bar is 50 µm. (**C**) Colocalization of GNAO1 variants (shown in green) with BDP TR ceramide (shown in red) in the Golgi apparatus (live confocal imaging, maximum intensity projection). Scale bar is 20 µm.

The clear difference in GNAO1 isoform expression patterns between astrocytes and neurons raised the question of possible functional differences between GNAO1-A and GNAO1-B. As previously reported, some mutations in GNAO1 can lead to changes in its intracellular localization [6,36]. We, therefore, hypothesized that the different GNAO1 isoforms, which differ by 20 amino acids, could also exhibit different localization patterns in both the WT and mutated forms. To test this hypothesis, we established a cell model to detect GNAO1 localization in astrocytes via live imaging. To generate astrocyte cultures stably expressing different GNAO1 variants fused to reporter proteins, we produced a set of lentiviral vectors (Table 4, Figure 3A, and Supplementary Figure S2). We selected the G203R variant of GNAO1 as a pathogenic allele for testing due to the specific symptom profile of G203R patients, who develop both epileptic encephalopathy and movement disorders. We used these vectors to infect IPSC-derived astrocytes from a healthy donor 2 (RCPCMi007-A). Since the lentiviral infection efficiency ranged from 90% to 100%, we did not perform cell sorting to separate infected from non-infected cells.

In astrocytes expressing GNAO1-A_WT, GNAO1-B_WT, GNAO1-A_G203R, or GNAO1-B_G203R, all GNAO1-GFP variants localized to the cell membrane and the Golgi apparatus (Figure 3B). Golgi localization was further confirmed by colocalization with BDP TR ceramide (Figure 3C). This localization pattern differed from that of control cells expressing GFP alone (which showed nuclear and cytoplasmic localization), allowing us to conclude that the observed localization was driven by the GNAO1 protein. This pattern was consistent with previous reports for other cell types [6]. These data demonstrated that there was no difference between GNAO1 isoforms in terms of intracellular localization in astrocytes.

### 3.4. GNAO1 Localized in Astrocytic Retraction Fibers

**Figure 4:**
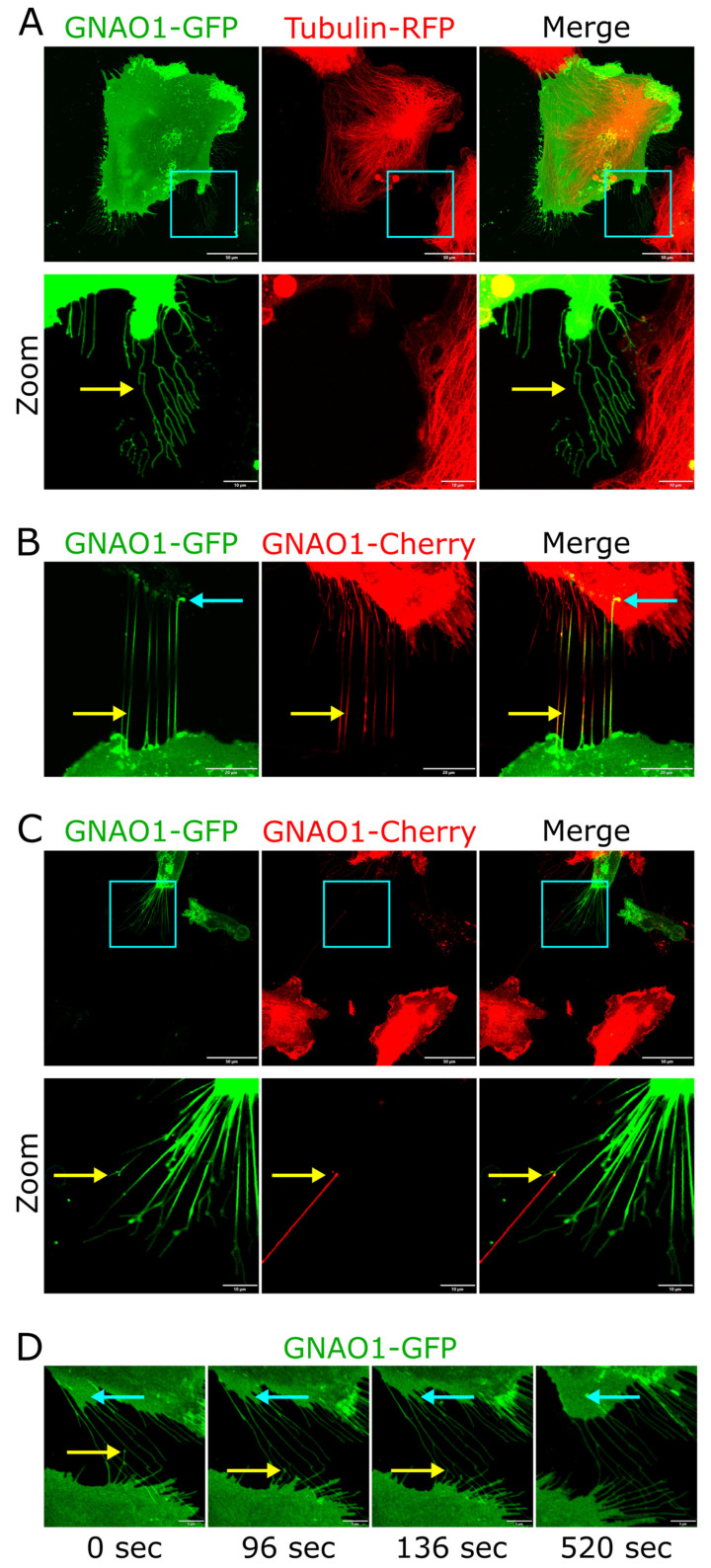
(**A**) Upper panel: An astrocyte expressing GNAO1-B_WT-GFP and RFP-α-tubulin (live confocal imaging, maximum intensity projection). Scale bar is 50 µm. Lower panel: Zoomed-in fragment of upper panel with adjusted LUTs. The yellow arrow indicates GNAO1-positive, tubulin-negative retraction fibers. Scale bar is 10 µm. (**B**) Astrocytes separately expressing GNAO1-GFP and GNAO1-Cherry (confocal imaging, maximum intensity projection). Blue arrows indicate retraction fiber contacts with a neighboring cell, and yellow arrows indicate tight antiparallel contacts between retraction fibers. Scale bar is 20 µm. (**C**) Upper panel: Astrocytes separately expressing GNAO1-GFP and GNAO1-Cherry (live confocal imaging, maximum intensity projection). Scale bar is 50 µm. Lower panel: Zoomed-in fragment of upper panel with adjusted LUTs. Yellow arrows indicate points of tip-to-tip contact between retraction fibers of neighboring astrocytes. Scale bar is 10 µm. (**D**) Time-lapse imaging of astrocytes expressing GNAO1-GFP (live confocal imaging). Yellow arrows indicate a moving tip of a retraction fiber attaching to a retraction fiber of a neighboring cell and maintaining a stable contact. The blue arrow indicates lamellipodia movement back along the retraction fibers. Scale bar is 5 µm.

For all GNAO1 variants, we found that GNAO1 localized to multiple 1 µm thin projections extending from the cell membrane of astrocytes. These projections emerged behind the leading edge during cell movement, lacked α-tubulin, and formed a characteristic network of fibers, allowing us to identify them as retraction fibers (Figure 4A). Retraction fibers are thin membrane extensions that link cells to the extracellular matrix and are formed during cell surface movement. They influence spindle orientation during mitosis and also play a role in cell migration by enabling faster movement through residual retraction fibers left by preceding cells [37,38,39]. Since GNAO1 localized to retraction fibers irrespective of isoform or mutational status, subsequent investigations focused on astrocytes expressing GNAO1-B_WT-GFP as a representative model.

As previously reported, retraction fibers facilitated the rapid return of migrating cells to their original position by enabling the fast movement of lamellipodia along these fibers (Figure 4D, Supplementary Video S2). Some retraction fibers lost contact with the substrate and migrated alongside the cell but could adhere to retraction fibers of neighboring cells (Figure 4D). We found no prior reports of retraction fibers in astrocytes, particularly of cell-to-cell contacts mediated by these structures. To develop a more advanced model for clearer visualization, we generated another set of lentiviral vectors encoding GNAO1 fused to Cherry. We then separately infected astrocytes with GNAO1-B_WT fused to Cherry or GFP and co-cultured them to enable simultaneous visualization. When two or more cells were in close proximity, some fibers terminated not on the substrate/matrix but on adjacent astrocytes. These contacts were stable and persisted even during cell movement (Figure 4B). Interestingly, we observed bidirectional contacts between the retraction fibers of adjacent cells. These anti-parallel fibers were tightly apposed along their entire length and moved coordinately (Figure 4B). Astrocytic retraction fibers also formed stable tip-to-tip contacts with retraction fibers from other cells. These connections extended up to 80 µm between migrating astrocytes (Figure 4C).

### 3.5. GNAO1 Localized in Astrocytic Migrasomes

**Figure 5:**
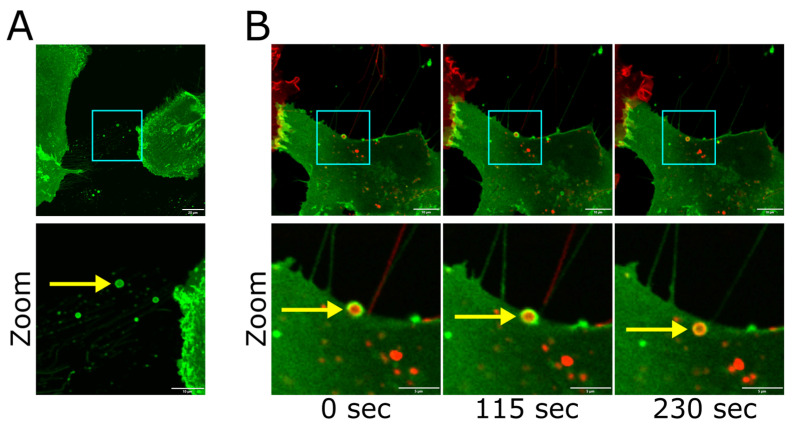
(**A**) Upper panel: Astrocytes expressing GNAO1-GFP (live confocal imaging, maximum intensity projection). Scale bar is 20 µm. Lower panel: Zoomed-in fragment of upper panel with adjusted LUTs. Yellow arrow indicates migrasome. Scale bar is 10 µm. (**B**) Upper panel: Time-lapse imaging of astrocytes separately expressing GNAO1-GFP and GNAO1-Cherry (live confocal imaging). Scale bar is 10 µm. Lower panel: Zoomed-in fragment of upper panel with adjusted LUTs. Yellow arrow indicates endocytosis of a GNAO1-Cherry-positive migrasome by GNAO1-GFP-positive astrocyte. This creates the appearance of a red structure (the migrasome) being enclosed by a green membrane (the acceptor cell). Punctate Cherry-positive inclusions (potentially internalized cellular fragments or digested vesicular material) are also visible in the recipient cell. Scale bar is 2 µm.

One of the accompanying elements of retraction fibers is migrasomes—extracellular vesicles formed on retraction fibers [40,41,42]. Migrasomes can transfer proteins from a migrating cell to neighboring cells [43]. We observed GNAO1-positive migrasomes in astrocytes (Figure 5A), which confirms the localization of GNAO1 in migrasomes [42]. As previously reported, migrasomes can be absorbed by neighboring cells. Indeed, in co-culture experiments of astrocytes expressing GNAO1-GFP or GNAO1-Cherry, we observed that migrasomes secreted by one astrocyte were absorbed by another astrocyte (Figure 5B).

Since GNAO1 is a component of the GPCR signaling cascade, which can initiate intracellular calcium waves, and because GNAO1 localizes to fibers that maintain cell-to-cell contact, we investigated whether retraction fibers could propagate calcium waves. We visualized calcium waves using FluoriCa-8 AM staining and observed calcium wave propagation in retraction fibers in both directions (Supplementary Video S3). Interestingly, calcium waves that emerged in the cell soma did not evoke calcium waves in all neighboring retraction fibers simultaneously but in different fibers at different times during imaging. We, therefore, hypothesized that retraction fibers may play a role in cell communications for both migrating and stationary cells and that GNAO1, as a key transducer of GPCR signals localized to these fibers, is involved in this process.

### 3.6. GNAO1-A Overexpression Reduced Astrocytic Calcium Activity

**Figure 6:**
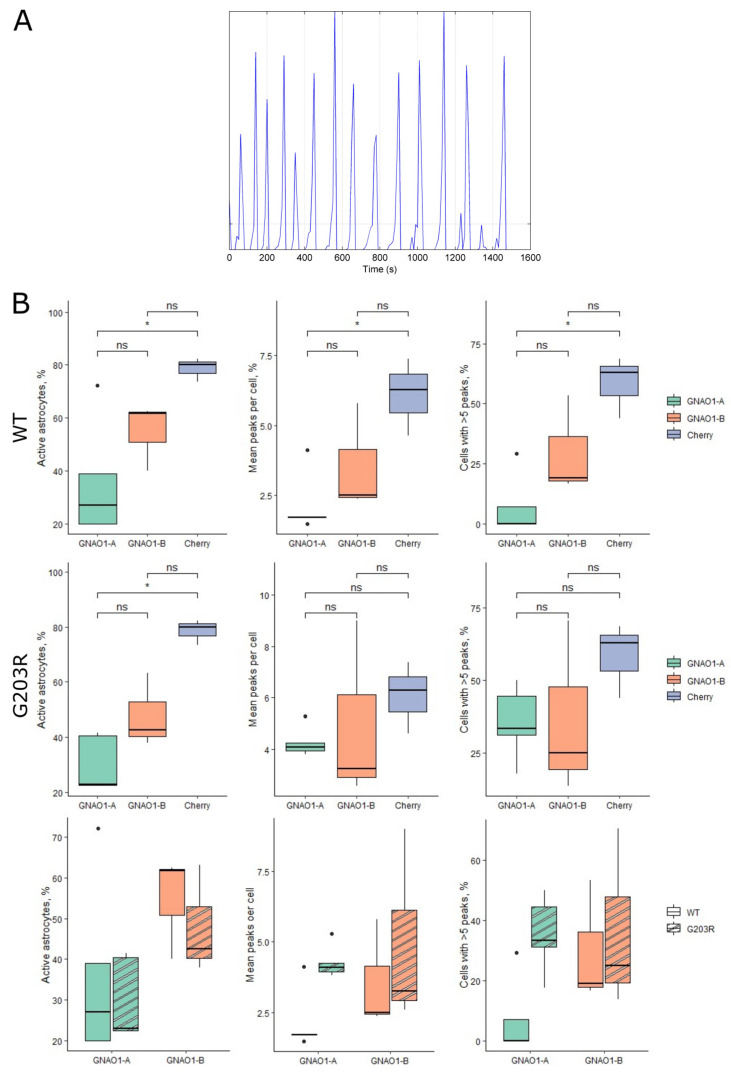
(**A**) Representative ΔF/F0 calcium traces for an astrocyte-expressing wild-type GNAO1-B. (**B**) Calcium activity of astrocytes overexpressing variants of GNAO1-Cherry or Cherry only. The upper row shows data for WT GNAO1 variants, the middle row shows data for G203R variants, and the lower row shows a comparison of WT and G203R variants. Whiskers represent 1.5 interquartile range of the data. Dots represent outliers. Significance levels: *—*p* < 0.05, ns—*p* > 0.05.

As mentioned previously, GNAO1 is involved in signal transduction from receptors and, therefore, participates in the initiation of calcium waves. We tested spontaneous calcium activity in astrocytes overexpressing variants of GNAO1-Cherry, using astrocytes expressing Cherry alone as a control. Not all astrocytes in the culture were able to generate calcium waves during the experiment. The cells that were active were divided into two groups based on the number of calcium waves they generated during the imaging period: cells with 1–4 peaks or cells with multiple peaks (Figure 6A). We measured the following parameters: the percentage of active cells, the mean number of calcium peaks per cell in active cells, and the percentage of active cells with five or more peaks during the imaging period (Figure 6B). Overexpression of both wild-type GNAO1-A and GNAO1-B reduced astrocytic calcium activity in all three parameters compared to astrocytes expressing Cherry only. GNAO1-A overexpression significantly (*p* < 0.05) reduced activity, whereas for GNAO1-B, statistical tests showed no significant differences from the control group, although a trend was visible on the corresponding graphs (Figure 6B). The G203R mutation in both GNAO1-A and GNAO1-B increased the mean number of calcium peaks per cell and the number of cells with five or more peaks. Overexpression of G203R-mutant GNAO1 slightly reduced the percentage of active cells, but this was not statistically significant.

## 4. Discussion

Taken together, these data demonstrate that GNAO1 functions in the human brain are not limited to neurons or Schwann cells but also involve astrocytes. Astrocytes exhibit a significantly high level of GNAO1 expression, suggesting their potential involvement in GNAO1-associated pathologies. More surprisingly, astrocytes show a stable, strict prevalence of GNAO1-B expression across donors. That raises two key questions for further investigation. First, it suggests that the GNAO1-B isoform, often considered less significant in the context of GNAO1 pathologies, could be the primary or even exclusive player in astrocyte dysfunction. Therefore, all GNAO1 mutations should be studied not only in the GNAO1-A genetic context but also in the GNAO1-B context. Second, what are the functional differences between GNAO1-A and GNAO1-B? We hypothesized that the difference could be in subcellular localization and, therefore, in the protein partner spectrum.

We did not detect a clear difference in the localization of GNAO1-A and GNAO1-B, either in the WT or G203R state. However, our study has several limitations. We focused on live-cell imaging, which offers numerous advantages but requires the use of a fluorescent reporter fusion. This fusion could potentially influence certain protein–protein interactions of GNAO1, particularly within the compact G-protein complex, as well as the localization of the protein within the cell. This potential effect might mask localization differences between native GNAO1-A and GNAO1-B. While our approach is consistent with previous studies investigating the influence of GNAO1 mutations on localization [12,44], further investigations are necessary to confirm the intracellular localization of these GNAO1 isoforms.

Here, we report for the first time the GNAO1 localization in retraction fibers. As a component of GPCR signaling machinery, GNAO1 may play a role in cell-to-cell communications via retraction fibers. We demonstrated that astrocytic retraction fibers can form contacts not only with the substrate but also with the membranes of other cells or retraction fibers. These fibers established stable intercellular connections that may enable rapid reversal of cell movement. Tight, stable, antiparallel, and tip-to-tip contacts between retraction fibers may allow migrating cells to maintain adhesion during locomotion. To our knowledge, we also provided the first evidence of Ca^2+^ wave propagation along retraction fibers.

Migrating cells communicate via migrasomes, which often contain a set of signaling molecules. Here, we report the localization of GNAO1 in astrocytic migrasomes and the transfer of GNAO1-positive migrasomes between astrocytes. In the context of the predominant expression of GNAO1-B in astrocytes, this finding raises the question of whether GNAO1-B is transferred from astrocytes to neurons via migrasomes, potentially altering the GNAO1 isoform ratio in neurons. We showed that the overexpression of both GNAO1-A and GNAO1-B reduced overall calcium activity in astrocytes. Calcium activity was nearly two times lower with GNAO1-A overexpression compared to the control. This finding is consistent with the very low endogenous expression of GNAO1-A in astrocytes. We propose that the astrocytic signaling system is optimized to operate with GNAO1-B and that GNAO1-A competes with GNAO1-B, thereby reducing overall activity. The G203R mutation slightly reduced the percentage of active cells in GNAO1-B-expressing cultures but had no significant influence on the mean number of peaks per cell or the number of cells with five or more peaks. We propose that, consistent with neuronal models, different GNAO1 mutations may have diverse effects on astrocytes. Previous studies have shown that astrocytes release several types of gliotransmitters upon activation, some of which can depress presynaptic neuronal activity [45,46]. As demonstrated by our data for wild-type GNAO1-A and GNAO1-B, different GNAO1 variants can lower astrocytic activation frequency, potentially impairing the synaptic depression process. This mechanism may be crucial in the context of epileptic syndrome onset and pathogenesis. As we show, GNAO1-B is the predominantly expressed isoform of GNAO1 in astrocytes. Astrocytes play an important role in synaptic function and are key components of the tripartite synapse alongside neurons. Therefore, GNAO1-B may play a significant role in signal transduction within the nervous system.

## 5. Conclusions

Astrocytes predominantly expressed the GNAO1-B isoform, whereas in GABAergic neurons, no significant difference was observed between GNAO1-A and GNAO1-B expression. The GNAO1 isoform ratio exhibited high stability in astrocytes but was highly variable in neurons. We found no difference between GNAO1-A and GNAO1-B localization in astrocytes, both in WT and G203R states. GNAO1 localized to retraction fibers and migrasomes. Retraction fibers participated in maintaining cell-to-cell contacts and also provided calcium waves during astrocytic excitation. GNAO1-positive migrasomes can be transferred between astrocytes. Overexpression of both GNAO1-A and GNAO1-B reduced calcium activity in astrocytes. We propose astrocytes as a new model for studying GNAO1-related pathologies and suggest that all GNAO1 mutations should be evaluated in the context of both GNAO1-A and GNAO1-B isoforms.

## Figures and Tables

**Table 1:** Cell line descriptions.

IPSC Line	Age, Sex, Race	Original Paper/IPSC Registry
Healthy donor 1	60 years, male, Caucasian	[26]
Healthy donor 2 (RCPCMi007-A)	48 years, female, Caucasian	[27] https://hpscreg.eu/cell-line/RCPCMi007-A (accessed on 7 November 2025)
Healthy donor 3	26 years, female, Caucasian	[28]
GNAO1 G203R patient	1 year, male, Caucasian	[11]

**Table 2:** PCR primers and probes.

Primer Name	Target Description	Primer Sequence	Melting T (°C), Product Size (bp)
GPO1	Mycoplasma detection	ACTCCTACGGGAGGCAGCAGTA	55, 705
MGSO		TGCACCATCTGTCACTCTGTTAACCTC	
GNAO1_Q_isoform_F	RT-qPCR with TaqMan-like probes	ATCTGAACGCAAGAAGTGGA	55, 254
GNAO1_Q_isoform_R		ATTCAGGAAAGCAGATGGT	
GNAO1_total_F	Amplification of total GNAO1 for RT-qPCR	CGGAGCAAGGCGATTGAGA	60, 88
GNAO1_total_R		ATTCTCCAGCCCCGAGCAG	
CDS_forward	Amplification of GNAO1 coding sequence	AACAAGATATCGCCACCATGGGATGTACTCTGAGCGCAG	72, 1065
CDS1_reverse		ACACAGATATCTCCGTACAAGCCGCAGCCCC	
CDS2_reverse		ACACAGATATCTCCGTAGAGTCCACAGCCCC	
Isoform1_FAM	GNAO1-A-specific probe	+GT+T+CTTCA+TCGATAC+CT (“+” stands for LNA nucleotide)	67
Isoform2_HEX	GNAO1-B-specific probe	A+ATGGTTCA+CAGA+CACGT (“+” stands for LNA nucleotide)	68

**Table 3:** List of antibodies used in ICC and WB.

Target/Type	Manufacturer/Catalogue Number	Dilution
MAP2	Invitrogen (Waltham, MA, USA), MA512823	1:200
TH	Abcam (Cambridge, UK), ab112	1:2000
GABA-A-rc-alpha2	Abcam (Cambridge, UK), ab176170	1:100
S100B	Abcam (Cambridge, UK), ab11178	1:200
GNAO1	ThermoFisher (Waltham, MA, USA), PA5-30044	1:1000 (for WB)
GAPDH	Hytest (Moscow, Russia), 5G4CC	1:1000 (for WB)
GFAP	Hytest (Moscow, Russia), 4G25	1:100
Alexa Fluor 488-conjugated anti-rabbit secondary antibody	Abcam (Cambridge, UK), ab181448	1:1000
Alexa Fluor 555-conjugated anti-mouse secondary antibody	ThermoFisher (Waltham, MA, USA), A21424	1:1000
HRP-conjugated anti-rabbit secondary antibody	Sigma-Aldrich (St.Louis, MO, USA), A9169	1:80,000 (for WB)
HRP-conjugated anti-mouse secondary antibody	Sigma-Aldrich (St.Louis, MO, USA), A9044	1:80,000 (for WB)

**Table 4:** Lentiviral vectors.

Lentiviral Vector Backbone	Gene of Interest	Fused Reporter Protein
LeGo-G2	GNAO1-A-WT	GFP
LeGo-G2	GNAO1-B-WT	GFP
LeGo-G2	GNAO1-A-G203R	GFP
LeGo-G2	GNAO1-B-G203R	GFP
LeGo-C2	GNAO1-A-WT	Cherry
LeGo-C2	GNAO1-B-WT	Cherry
LeGo-C2	GNAO1-A-G203R	Cherry
LeGo-C2	GNAO1-B-G203R	Cherry
LeGo	Alpha-tubulin	RFP

## Data Availability

The original contributions presented in this study are included in the article/Supplementary Material. Further inquiries can be directed to the corresponding author(s).

## Supplementary Materials

The following supporting information can be downloaded at https://www.mdpi.com/article/10.3390/cells14221755/s1: Figure S1: Anti-GFAP staining of generated astrocytes and anti-TH and anti-S100B staining of generated neurons. Figure S2: Western blot. Video S1: Calcium oscillations in generated astrocytes. Video S2: Retraction fibers form contacts during movement. Video S3: Calcium waves in retraction fibers.

## Abbreviations

The following abbreviations are used in this manuscript:

AA	Amino acid
BDNF	Brain-derived neurotrophic factor
cDNA	Complementary DNA
CDS	Coding sequence
FBS	Fetal bovine serum
GDNF	Glia-derived neurotrophic factor
GPCR	G-protein-coupled receptor
hESC	Human embryonic stem cells
IPSC	Induced pluripotent stem cells
NEAA	Non-essential amino acids
P/S	Penicillin–streptomycin
PBS	Phosphate-buffered saline
SR	Serum replacement
UTR	Untranslated region
WT	Wild type
