# Supplementary material for: The GNAO1-B Splice Variant Is the Predominant Isoform in Human Astrocytes and Localizes to Retraction Fibers and Migrasomes
Source: Cells. 2025 Nov 10;14(22):1755. doi: 10.3390/cells14221755 (PMC12651897; doi:10.3390/cells14221755)
Supplement: Supplementary file 1 [file cells-14-01755-s001.zip › Supplementary-cells-3900928.pdf]

The GNAO1-B splice variant is the predominant isoform in human astrocytes and localizes to retraction fibers and migrasomes

Egor Volovikov[1,*], Alina Davidenko[1], Elizaveta Emets[1,2], Anastasia Smirnova[3,4], Alexandra Bogomazova[1] and Maria Lagarkova[1]

1 Lopukhin Federal Research and Clinical Center of Physical-Chemical Medicine of Federal Medical Biological Agency, 119435 Moscow, Russia
2 Federal State Budgetary Institution of Science "M.M. Krasnov Research Institute of Eye Diseases" (Krasnov Research Institute of Eye Diseases), 119021 Moscow, Russia
3 Centre for Regenerative Medicine, Medical Research and Education Institute, Lomonosov Moscow State University, Moscow, 119234, Russia
4 Faculty of Medicine, Medical Research and Education Institute, Lomonosov Moscow State University, Moscow, 119234, Russia

* Correspondence: volovikovea@rcpcm.org

Supplementary materials

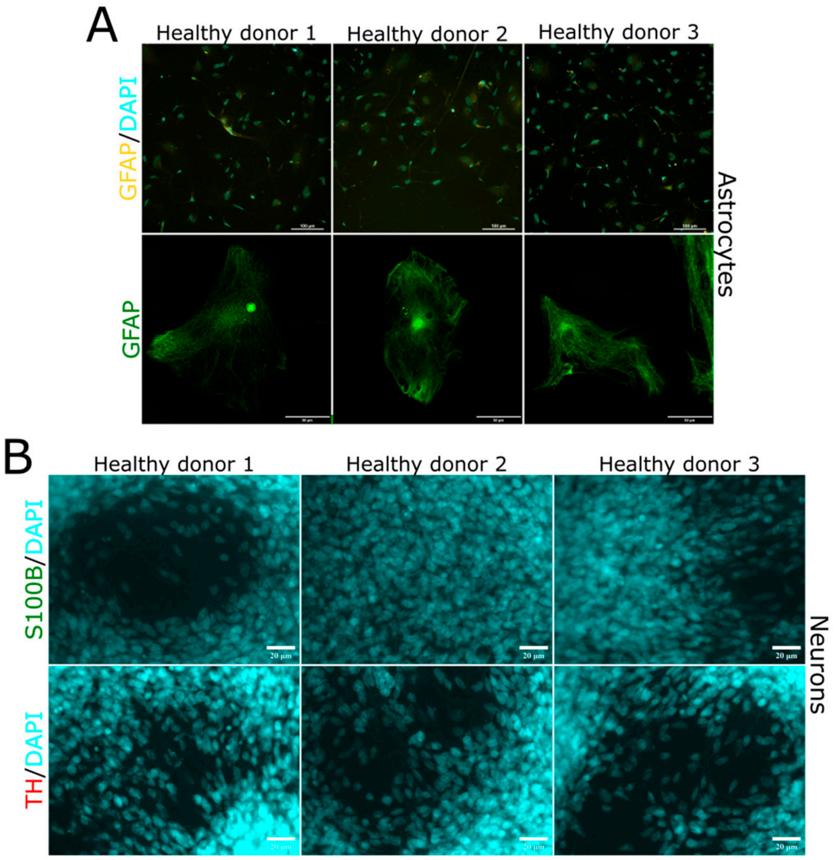

Supplementary Figure S1. **(a)** - Immunocytochemical staining of the generated astrocytes. Astrocytes expressed GFAP, a specific astrocytic marker. Upper row – widefiled microscopy, scale bar is 100 µm. Lower row – confocal microscopy, maximum intensity projection. Scale bar is 50 µm. **(b)** -

Immunocytochemical staining of the generated neurons. Neurons show no significant expression of S100B and TH. Widefield microscopy, scale bar is 20 μm.

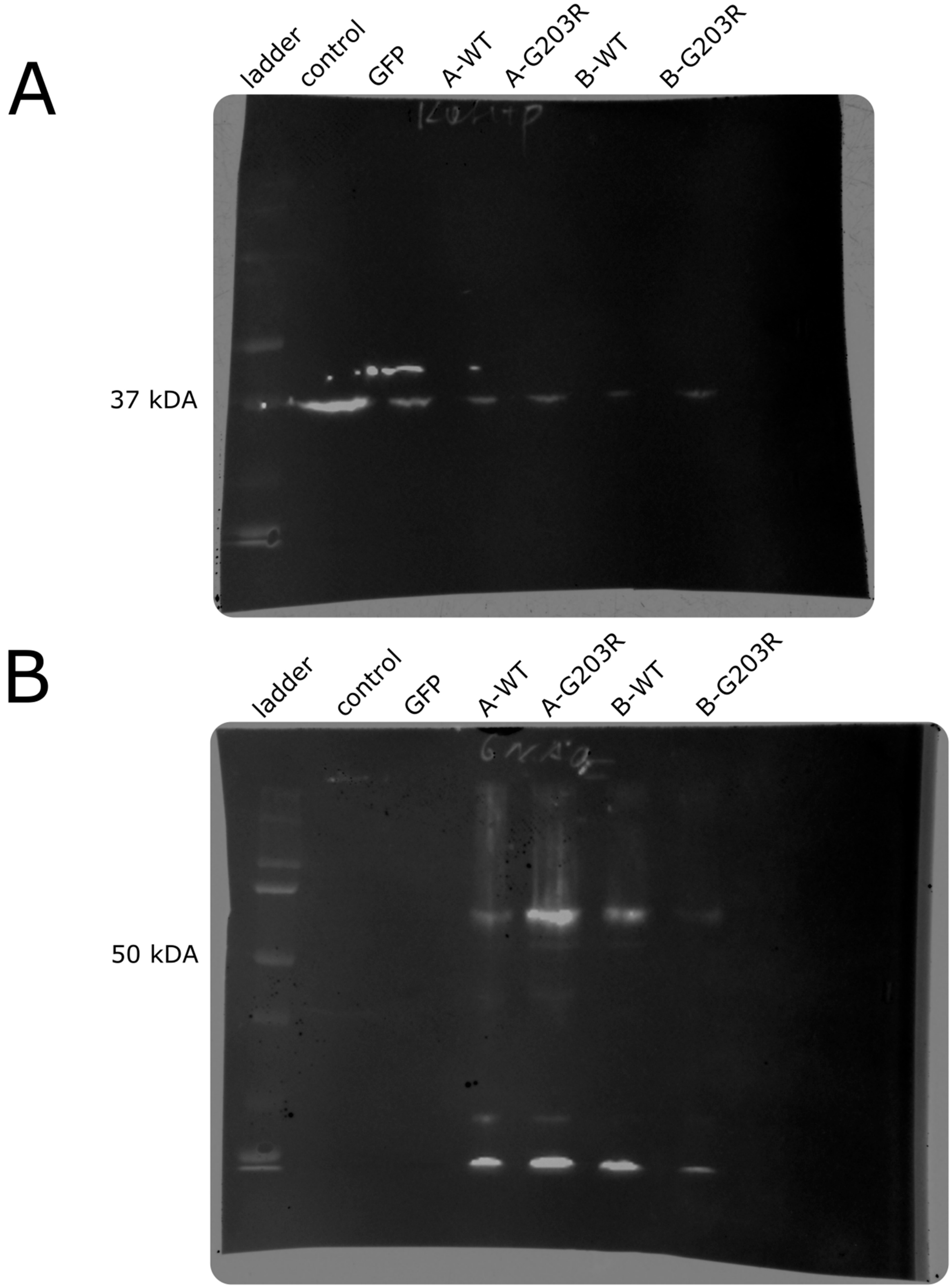

Supplementary Figure S2. Uncropped western blot images of HEK293 cell lysates. (A) Staining for GAPDH. (B) Staining for GNAO1. "Control" refers to untreated HEK293 cells; "GFP" refers to HEK293

cells expressing GFP only. "A-WT", "A-G203R", "B-WT", and "B-G203R" denote HEK293 cells expressing the respective GNAO1 variants fused to GFP. The molecular weight ladder is the Kaleidoscope Prestained Protein Standard.

Supplementary Video S1. Representative time-lapse imaging of live astrocytes stained with FluoriCa-8 AM (green). Calcium waves oscillation and propagation between cells can be seen. Timestamp: mm:ss.

Supplementary Video S2. Time-lapse imaging of live astrocytes expressing wild-type GNAO1-B fused to GFP. Retraction fibers, located in the center, can be seen forming new contacts with each other during cell movement. The focus in the upper part of the imaging area is on a migrating astrocyte exhibiting reverse movement, with its membrane spreading along the retraction fibers. Timestamp: hh:mm:ss.

Supplementary Video S3. Time-lapse imaging of live astrocytes expressing wild-type GNAO1-B fused to Cherry (not visible) and stained with FluoriCa-8 AM (green). A network of retraction fibers is located to the right of the lower astrocyte. Propagation of calcium waves along the retraction fibers is visible in both directions—toward the extracellular matrix and the neighboring astrocyte. The retraction fibers do not propagate calcium waves simultaneously but show distinct initiation time points. Timestamp: hh:mm:ss.
